# Lysophosphatidic Acid (LPA) Signaling in Human and Ruminant Reproductive Tract

**DOI:** 10.1155/2014/649702

**Published:** 2014-03-12

**Authors:** Izabela Wocławek-Potocka, Paulina Rawińska, Ilona Kowalczyk-Zieba, Dorota Boruszewska, Emilia Sinderewicz, Tomasz Waśniewski, Dariusz Jan Skarzynski

**Affiliations:** ^1^Department of Reproductive Immunology and Pathology, Institute of Animal Reproduction and Food Research, Polish Academy of Sciences, Tuwima 10 Street, 10-747 Olsztyn, Poland; ^2^Department of Gynecology and Obstetrics, Faculty of Medical Sciences, University of Warmia and Masuria, Zolnierska 14 C Street, 10-561 Olsztyn, Poland

## Abstract

Lysophosphatidic acid (LPA) through activating its G protein-coupled receptors (LPAR 1–6) exerts diverse cellular effects that in turn influence several physiological processes including reproductive function of the female. Studies in various species of animals and also in humans have identified important roles for the receptor-mediated LPA signaling in multiple aspects of human and animal reproductive tract function. These aspects range from ovarian and uterine function, estrous cycle regulation, early embryo development, embryo implantation, decidualization to pregnancy maintenance and parturition. LPA signaling can also have pathological consequences, influencing aspects of endometriosis and reproductive tissue associated tumors. The review describes recent progress in LPA signaling research relevant to human and ruminant reproduction, pointing at the cow as a relevant model to study LPA influence on the human reproductive performance.

## 1. Introduction

The present paper focuses particularly on one of the simplest and most potent lysophospholipids, lysophosphatidic acid (LPA), and summarizes recent knowledge on its biological impact on human and ruminant reproduction.

## 2. Lysophosphatidic Acid Production and Receptor-Mediated Signaling

Lysophosphatidic acid is a simple phospholipid that exerts many physiological and pathological actions on various cell types, such as cell proliferation and differentiation [1], cytoskeletal rearrangement [2], cell-to-cell interactions [3], and tumorigenesis [4].

So far LPA has been detected in many various biological fluids such as serum and plasma [5–7], tears [8], ascites [9], seminal plasma [10], and follicular fluid [11]. Moreover, it can also be produced in various cell types like endometrial cells [12, 13], ovarian cells [12, 14–16], mast cells [17], erythrocytes [18], neurons [19], and many others [20].

While the precise mechanism of LPA metabolism within most types of cells is still unclear, two general pathways of LPA production have been demonstrated. In the first pathway phosphatidic acid (PA) is produced from phospholipids (PLs) by phospholipase D (PLD), also called autotaxin (ATX) or from diacylglycerol by diacylglycerol kinase [21]. In both pathways there is deacylation of PA to LPA by phospholipase (PLA)-type enzymes. In the second pathway, PLs are first converted to lysophospholipids (LPLs) by the action of secretory (sPLA_2_), PS-PLA_1_, and lecithin-cholesterol acyltransferase (LCAT), and then the LPL is converted to LPA by ATX [22]. The first pathway is mainly involved in cellular LPA production, while the second pathway is involved in LPA production in extracellular body fluids, especially in serum and plasma. These various ways of LPA synthesis reflect multiple levels of regulation—or deregulation in the organism being at different physiological or pathological status—cancers [4], pregnancy [23], hypertension [24], prostate disease, or obesity [25]. Moreover, LPA-dependent different signaling pathways have clear therapeutic repercussions since pharmaceutical drugs targeting certain enzymes would differ from those targeting other LPA biosynthetic pathways [26, 27].

In mammals, LPA exerts its action via at least six high affinity, transmembrane G-protein-coupled receptor (GPCR) types, LPAR1–LPAR6, and possibly through a nuclear receptor PPAR*γ* [22, 28–31]. These LPARs are expressed in various organs and cells [21]. For example, LPAR1 is highly expressed in the nervous system [32], LPAR2 in immune organs such as the thymus and spleen [33], and LPAR3 in reproductive organs such as the ovary and uterus [7, 16, 34, 35]. On the other hand, LPAR4, LPAR5, and LPAR6 are expressed widely but at relatively low levels. In that aspect we can find LPAR4 expression in the ovary [30], LPAR5 expression in the small intestine, spleen, dorsal root ganglion, and embryonic stem cells [36]. However, there is also much evidence of an aberrant expression of LPA receptors in certain diseases, meaning especially different types of cancer [37, 38].

The influence of LPA on the reproductive system function of the female has been examined and described for about 30 years. Since the first reports published by Jarvis et al. [39] in women, various abnormalities in reproductive performance on different regulatory levels due to LPA signaling and LPARs knockout have been also reported in many farm animals including ruminants [34, 35, 40].

## 3. Effects of Lysophosphatidic Acid on the Reproductive Performance in Human

### 3.1. The Possibility of LPA Synthesis and LPARs Expression in the Reproductive Tissues

Physiologically, LPA and its active LPARs have been documented to be present in female reproductive organs, such as uterus [20, 41, 42], ovary [43, 44], and placenta [43, 45, 46] as well as in the amnion-derived cells* in vitro* [47]. Interestingly serum ATX level was higher in women than in men [48], suggesting possible influence of LPA on the female reproduction.

### 3.2. LPA Signaling in the Human Ovary

LPA signaling has been extensively studied in the physiology of human ovary. Follicular fluid of the human preovulatory follicle contains lysophospholipase D—ATX which is responsible for local LPA production from lysophosphatidylcholine (LPCs) [49]. However LPA in ovaries is produced not only from follicular fluid's LPCs, but also from LPCs in granulosa cells and oocytes. In follicular fluid taken from women programmed for* in vitro* fertilization the amount of LPA increases with the time of incubation with ATX in 37°C [11]. Serum ATX activity from patients receiving ovarian stimulation was higher than in women with natural cycles [44]. Moreover, Chen et al. [44] demonstrated mRNA expression of three LPA receptors, LPAR1, LPAR2, and LPAR3, in the granulosa-lutein cells from women undergoing* in vitro* fertilization. LPAR4 was also highly expressed in the cortex of the human ovary [30].

LPA signaling plays many crucial roles in ovarian function such as ovulation, for example. Before ovulation LPA elevated the level of IL-8, expressed its chemotactic activity for neutrophils, started the inflammatory reaction, and in consequence led to tissue degradation and rupture of the follicle [50]. During the luteal phase human ovary exhibits complete tissue remodeling with the stages of growth, differentiation, and regression [51]. In the early luteal phase granulosa and theca cells form corpus luteum with high level of angiogenesis. LPA through the upregulation of IL-8 and IL-6 stimulated the multistep process of new vessels formation in the CL [42]. Moreover, LPA induced expression of angiogenic cytokines, IL-6, and IL-8, in granulosa-lutein cells from women undergoing* in vitro* fertilization [42]. Thus, the authors concluded that the induction of these cytokines by LPA, through its receptor and nuclear factor-kappaB-dependent pathway, in the stimulated ovaries may contribute to ovarian hyperstimulation syndrome [42].

### 3.3. LPA Signaling during Pregnancy

More than a decade ago Tokumura et al. [49] suggested the involvement of LPA signaling in maintenance of human pregnancy. These authors demonstrated increasing levels of LPA and serum ATX/lysoPLD activity during pregnancy [49]. The elevated levels of serum LPA found during pregnancy were reported to arise from both the placenta and the fetus [49]. In the human placenta LPA can be produced locally by trophoblasts ATX and thereby control trophoblast proliferation, differentiation, feto-maternal immune interaction, and placental vascular remodeling [46]. During early pregnancy, placenta produces many new vessels which are crucial for fetal-maternal exchange. LPA, through induction of IL-8, could stimulate the process of angiogenesis in the placenta [45]. Kim et al. [47] also found that LPA modulated cellular activity and stimulated proliferation of human amnion cells* in vitro*. Moreover, Jarvis et al. [39] documented high lysoPLD activity in human placental tissues, with the highest in the amnion. The authors suggested that high lysoPLD activity in the amnion proclaimed that LPA might be involved in the regulation of labor, due to the direct implications of this membrane in the initiation of the labor [39]. Moreover, Jeng et al. [52] demonstrated that LPA upregulated transcriptional activity of oxytocin (OT) receptors* in vitro* which resulted in sensitization of myometrial cells to OT. In uterus in the end of gestation the sensitivity of myometrium to OT increases robustly, which in turn induces uterine contractions leading to labor. Other important process in induction and maintenance of smooth muscle contraction is stress fiber formation. LPA enhanced stress fiber formation in human myometrial cells* in vitro* and thereby increased the efficiency of uterine contractions in the beginning of labor [53].

The process of embryo implantation includes interaction between the endometrium and the embryo (adhesion and invasion), inflow of innate immune cells, and vascularization. LPA has been presented to be involved in implantation on many various levels. In human decidual cells, LPA increased embryo outgrowth as the result of RhoA signaling [54]. It also stimulated chemotaxis of NK cells and monocytes by inducing the transcription of MCP-1 and GRO-*α*, respectively, and thereby contributed to regulation of the innate immunity of the fetus [45]. Iwasawa et al. [46] documented that LPA activated lymphocytes and dendritic cells that induced inflammatory reaction which is essential in the process of implantation. Moreover, in an* in vitro* model of embryo implantation LPA increased the outgrowth of embryos on decidual cells [54]. Through IL-8 stimulation LPA participated in new vessels formation around the embryo [45].

The pleiotropic roles of LPA in the function of the reproductive tract are demonstrated not only by the increased amount of LPA in body fluids and in the area of reproductive organs but also by the tissue-specific, regulated expression of its receptors. Wei et al. [55] demonstrated that LPAR3 levels decreased in the middle and late secretory endometrium (when the implantation occurs) of women with endometriosis. Decreased LPAR3 expression was correlated with the expression of other uterine receptivity biomarkers, such as osteopontin or HOXA10, all regulated by progesterone (P4) [55]. The authors claimed that reduced expression of these genes may explain P4 resistance associated with endometriosis [55].

LPA signaling can also have adverse effects during pregnancy. Li et al. [56] detected high levels of LPAR2 and LPAR3 gene expression in the placentas of patients with gestational hypertension and preeclampsia. Moreover, it has been documented that LPA elevated arterial blood pressure [57] and vasoconstriction [58]. Taking into consideration, increasing levels of LPA during pregnancy [49] and LPA involvement in elevating blood pressure, we might suppose the adverse effects of this lipid in the terminating stages of pregnancy. In addition, accumulation of LPA in blood contributed to platelet-monocyte coaggregate formation [59] as well as enhanced platelet aggregation and adhesion [60] which in turn might have resulted in thrombosis during pregnancy. Tokumura et al. [61] also presented the direct association of elevated levels of LPA in blood circulation with induction and/or progression of systemic vascular dysfunction seen in patients with preterm labor or preeclampsia. On the other hand, LPA might also contribute indirectly to infection-related preterm labor via the induction of arachidonic acid (AA) metabolites. Mikamo et al. [62] presented elevated levels of LPC, the substrate for LPA, and AA in human uterine endometrial cells exposed to extracts from pathogens involved in intrauterine infection.

### 3.4. LPA Signaling in the Reproductive Tissue Associated Tumors and Other Disorders of Reproductive Functions

LPA signaling may also play a role in pathogenesis of both benign and malignant endometrial tumors. Billon-Denis et al. [63] presented LPA influence on the growth of leiomyomas or fibroids. Treatment of leiomyoma tumor-derived cell line with LPA entailed DNA synthesis through ERK activation [63]. The authors also proposed that LPA produced in leiomyomas* in vivo* may have been involved in tumor cell proliferation [63]. There are also reports indicating that LPA promoted endometrial cancer invasion via the induction of matrix metalloproteinase-7 (MMP-7) [64, 65]. Rapizzi et al. [66] used the cervical cancer cell line, HeLa, to study the roles of LPA in cervical cancer. In these studies, LPA induced HeLa cell migration and survival [66].

LPA signaling may also play a role in breast cancer progression. Kitayama et al. [67] suggested the involvement of the upregulation of LPAR2, but not LPAR1 or LPAR3, in the mammary gland carcinoma pathogenesis in the breast cancer of postmenopausal women. Other studies from breast cancer cell lines indicated that both LPAR1 and LPAR2 mediated LPA-induced chemotaxis in breast carcinoma cells [68]. LPA signaling was also involved in breast cancer cell proliferation [69]. Boerner et al. [70] demonstrated that LPA-dependent overexpression of EGF receptor (a prognostic indicator of a poor outcome in tumors) was involved in breast cancer progression [70].

Ovarian cancer is the most thoroughly studied cancer with respect to LPA signaling in carcinogenesis. There are two types of data in the literature, demonstrating the direct and indirect roles of LPA in the pathogenesis of the tumors. Sutphen et al. [71] and Xu et al. [72] demonstrated that elevated levels of LPA in the plasma and ascites of ovarian cancer patients, promoted ovarian cancer cell proliferation. LPA signaling may also exert its role in ovarian cancers indirectly through regulating telomerase, involved in tumor progress [73], IL-6 and IL-8 involved in tumor angiogenesis [74], or COX-2, correlated with possibility of metastasis [75].

Moreover, the regulation of LPARs may also play a significant role in LPA signaling in ovarian cancer. Wang et al. [76] proved that LPAR2 and LPAR3 were upregulated in ovarian cancer tissues. Sengupta et al. [77] demonstrated that LPAR3 was a key receptor for mediating the chemotactic activity of LPA in ovarian tumors. On the other hand, LPAR1 was demonstrated to be the key receptor in mediating ascitic LPA effects on other cells [78].

Overlooking the available data in the literature on the role of LPA in human reproduction we can summarize that the past decade shaded new light on the importance of LPA signaling not only under physiological but also pathological conditions. LPA is produced locally in human reproductive tissues and controls ovarian cycle and pregnancy as well as various abnormalities in the female reproductive tract. Engagement of LPA in the maintenance of pregnancy manifests by its action on the uterus, ovary, fetal membranes, and placenta. LPA can also affect the fetus itself by controlling the process of implantation and vascularization of the embryo. However, there are also reports on adverse effects of LPA in the female reproduction, especially during tumorigenesis. Since in the available literature there are often registered opposite effects of LPA on the reproductive performance in human as well as the research on human tissues is ethically restricted and new LPA-targeted therapeutic strategies are demanded, it is important to find a good animal model to study LPA influence on the function of the female reproductive organs.

## 4. Relevance of a Cow Model to Human Reproductive Performance 

Properly designed studies to examine the effects of LPA on the reproductive performance in humans should be done in human subjects. However, this is very hard to accomplish, since human studies are difficult to carry out because of their typical complexity and dependence on mostly retrospective data rather than the treatment-based outcomes measured in animal models including the bovine one. Moreover, all the complications in the design and interpretation of human studies, combined with the ethical issues regarding experimentation in humans, continuously increase interest in studies that utilize animal models. On the other hand, the relevance of studies performed in animal models to human health has been questioned many times in the literature, since in almost all animals used as a model many weak points can be found. Taking above arguments into consideration, it has been well documented in the literature that the cow can be one of the quite relevant animal models for studying human reproduction. In the bovine reproduction we can find many similar aspects in the ovarian physiology, early embryo development, pregnancy as well as assisted reproductive techniques [79, 80]. Therefore, we believe that the cow model has broad applicability and may be used to extend investigations to different physiologic/pathologic states and to other species including humans. Moreover, the bovine model ensures a greater availability of biological material compared to studies in human.

## 5. Effects of Lysophosphatidic Acid on the Reproductive Performance in Ruminants

### 5.1. The Possibility of LPA Synthesis and LPARs Expression in the Reproductive Tissues

**Figure 1:**
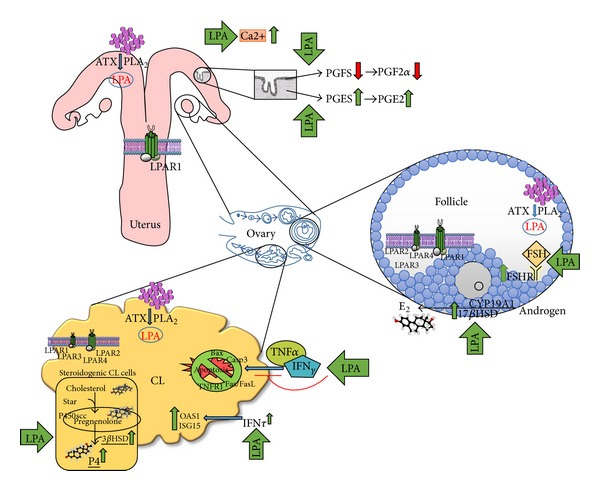
Schematic model illustrating the possible lysophosphatidic acid signaling in the bovine reproductive tract (LPA—lysophosphatidic acid; LPAR—lysophosphatidic acid receptor; PGFS—prostaglandin F_2*α*_ synthase; PGES—prostaglandin E_2_ synthase; ATX—autotaxin; PLA_2_—phospholipase A_2_; FSH—follicle stimulating hormone; FSHR—follicle stimulating hormone receptor; E_2_—estradiol; CYP19A1—cytochrome P450 aromatase; 17*β*HSD—17*β*-hydroxysteroid dehydrogenase; TNF *α*—tumor necrosis factor *α*; TNFR1—tumor necrosis factor *α* receptor type 1; IFN*γ*—interferon *γ*; IFN*τ*—interferon *τ*; Casp3—caspase 3; Fas/FasL—Fas antigen/Fas ligand; OAS1—2,5′-oligoadenylate synthase; ISG15—ubiquitin-like IFN-stimulated gene 15 kDa protein; StAR—StAR protein; P450 scc—cytochrome P450; 3*β*HSD—3*β*-hydroxysteroid dehydrogenase; PGF_2*α*_—prostaglandin F_2*α*_; PGE_2_—prostaglandin E_2_; and P4—progesterone). LPA derived from the blood plasma and produced in the uterus and ovary induces auto- and paracrine actions on the bovine endometrium, corpus luteum (CL), and the follicle. In the bovine endometrium LPA acting via LPAR1 induces PGE_2_ and inhibits PGF_2*α*_ actions. In the ovarian follicle, LPA stimulates E_2_ production and FSH action in granulosa cells via increased expression of the FSHR and 17*β*-HSD. In the bovine CL, LPA stimulates P4 secretion through stimulation of 3*β*HSD. LPA augments IFN*τ*-dependent stimulation of ISG15 and OAS1 expression in the steroidogenic cells of the bovine CL. LPA suppresses TNF*α* and IFN*γ*, induced luteal cell apoptosis via inhibition of the stimulatory effect of the cytokines on the expression of Bax, Fas—FasL system, TNFR1, and Casp3 activity in the cultured steroidogenic luteal cells, which orientates the cells towards the survival state.

In the bovine ovary, Boruszewska et al. [81] demonstrated ATX and PLA2 expression in bovine granulosa cells, which documented the possibility of LPA synthesis in bovine follicles, with ATX playing the major role in this process (Figure 1). LPA was also detected in picomole concentrations in the bovine CL throughout the estrous cycle and early pregnancy [16]. The concentration of LPA in the CL increased from days 2–4 to days 17–19 of the estrous cycle and during the estrous cycle was significantly higher than during early pregnancy [16]. The detected presence of LPA as well as enzymes responsible for LPA synthesis and specific LPARs in the CL tissue and the follicle indicate that bovine ovary can be a site of LPA synthesis during the estrous cycle and early pregnancy [7, 16, 81].

In ruminant uterus, the influence of LPA on the endometrial function was studied for the first time by Liszewska et al. [34] in sheep. This study revealed the involvement of LPA signaling in early embryo development. The authors found increased levels of LPA in ovine uterus at the time of early pregnancy, suggesting that LPA signaling contributed to the cross-talk between mother and embryo at the beginning of gestation [34].

In the cow, we were the first to demonstrate that LPA is locally produced and released from bovine endometrium during estrous cycle and early pregnancy [7]. We found significantly higher concentration of LPA in the blood taken from uterine vein on days 17–19 of the estrous cycle than in the blood from jugular vein. Moreover, we found high concentrations of LPA in the endometrial tissue [7]. LPA concentration in the bovine endometrium did not differ either during estrous cycle or early pregnancy (before implantation); however, it was significantly higher on days 17–19 of pregnancy than on days 17–19 of the estrous cycle.

Studying intracellular localization of the enzymes responsible for endometrial LPA synthesis, we demonstrated that ATX and PLA_2_ were immunoexpressed both in epithelial and stromal cells [82, Figure 1]. Boruszewska et al. [82] also found that LPA concentration was significantly higher in epithelial than instromal cells and ATX and PLA_2_ expression was higher in epithelial than instromal cells in bovine endometrium. This study demonstrated that epithelial cells are the main source of LPA in the bovine endometrium [82]. Similarly, in sheep, Liszewska et al. [34] found apical localization of ATX in glandular and luminal cells of endometrium. The authors also found that ATX activity was orientated towards the uterine lumen. Liszewska et al. [34] also documented that ATX level was 4- to 5-fold higher in the ovine uterus than in the trophectoderm. The authors postulated that LPA detected in the ovine uterus may be due to ATX activity on the maternal side [34]. However, in ovine conceptuses elevating expression of ATX was also demonstrated, which documented that the trophectoderm may also contribute to the production of LPA during pregnancy [34]. The data obtained in cows and sheep strongly suggest thatepithelial cells of the bovine endometrium are the main source of LPA inthese species. However, a supporting role of stromal cells in LPA synthesis cannot be excluded.

The potential role of LPA depends on both its local concentration and the distribution of LPARs in the area of reproductive tissues. In the bovine follicle, Boruszewska et al. [81] detected all types of LPARs at mRNA level in granulosa cells (Figure 1). However, LPAR1 transcript abundance was approximately 16- to 23-fold higher than expression of LPAR2, LPAR3, and LPAR4 mRNA [81]. In the bovine CL four types of LPARs were detected both during the estrous cycle and early pregnancy [16]. However, of the four LPARs examined, LPAR2 and LPAR4 were expressed the most strongly in the bovine CL [16, Figure 1]. In the bovine CL, high expression of LPAR4 compared with the other receptors during the estrous cycle and early pregnancy, as well as the dynamic changes of LPAR2 and LPAR4 during early pregnancy, probably accounts for the contribution of LPA to different events during the estrous cycle and pregnancy, namely, the contribution to P4 secretion, modulation of interferon (IFN)*τ* action during early pregnancy [16], or modulation of luteolysing cytokines action in the CL at the late luteal stage [83]. The data obtained by Kowalczyk-Zieba et al. [16] in the bovine CL do not completely agree with the results of Budnik and Brunswig-Spickenheier [84], who showed that LPA exerted its actions on bovine luteal cells only via LPAR2. The mRNA expression of all LPARs in granulosa cells as well luteal cells indicates that the bovine follicle and the CL represents a target for LPA action in the bovine reproductive system.

In sheep, Liszewska et al. [85] demonstrated that the expression of LPAR1 and LPAR3 in the ovine endometrium was regulated according to the estrous cycle. On the other hand, at day 12 of pregnancy, expression of both LPAR1 and LPAR3 in the endometrium was significantly reduced in comparison with day 12 of the estrous cycle [85]. The authors supposed that the decrease of LPARs expression in the endometrium was the result of the beginning of rapid growth and elongation of the ovine embryo, as well as modulation by various factors from conceptus origin [85]. However, in the ovine trophectoderm during the peri-implantation period LPAR1 and LPAR3 expression was the most abundant at the time of embryo implantation [34]. Moreover, Liszewska et al. [34] demonstrated perinuclear/nuclear and membrane localizations of LPAR1 in ovine conceptuses and trophectoderm cells cultivated* in vitro*, whereas LPAR3 was found only in the cell membrane in both systems. In the ovine uterus, LPAR1 was predominantly present in the stromal tissue, whereas LPAR3 was mostly detected in the epithelial structures [85]. In the bovine endometrial tissue only LPAR1 expression was detected [7]. LPAR1 expression increased from early to late luteal stage of the estrous cycle and reached the highest level at late luteal stage and on days 17–19 of early pregnancy [7]. On the other hand, LPA1 expression on days 8–10 of pregnancy was lower than that on days 17–19 of pregnancy but higher than on days 8–10 of the estrous cycle [7]. Boruszewska et al. [82] found higher LPAR1 expression in stromal than in epithelial cells. These results are in agreement with the fact that LPA on days 8–10 and 16–18 of the estrous cycle and early pregnancy stimulated prostaglandin (PG) E_2_ synthesis only in the* in vitro* cultured stromal cells [13, 40]. The overall results suggest that LPA in bovine endometrium is produced mainly by epithelial cells and affects mostly stromal cells acting* via* LPAR1.

Studying receptor and intracellular mechanism of LPA action in the ovine trophectoderm cells, Liszewska et al. [34] found that LPA stimulated the phosphorylation of ERK1/2* in vitro* and a specific antagonist of LPAR1 and LPAR3 receptors (VPC32183) blocked this effect. This study directly evidenced that LPARs operate and are functionally coupled to signal transduction mechanisms in trophectoderm cells [34]. In other cell types, the activation of ERK1/2 accounts for the proliferative effect of LPA [86]. Therefore, Liszewska et al. [34] claim that LPA amongst other factors in the uterus may be involved in the elongation of the conceptus during the peri-implantation period in the sheep as well as in mediating the cellular differentiation required for ovine embryo implantation. Liszewska et al. [34, 85] also reported that LPA stimulated changes in the organization of actin and tubulin architecture in ovine trophectoderm cells as well as in uterine epithelial cells* in vitro*. Therefore the authors suggested that LPA may be involved in the mechanisms regulating morphological changes both in the embryo and the uterus during conceptus adhesion to the uterus in ewes during the implantation process Liszewska et al. [34, 85].

The studies concerning receptor and intracellular mechanisms of LPA action in the bovine endometrial cells revealed that LPA stimulated PGE_2_ production, cell viability, and intracellular calcium ion mobilization in the cultured stromal endometrial cells via LPAR1 receptor activation [87].

In ruminants, the dynamic LPA synthesis and LPARs expression and action in the follicle, CL and uterus suggest that LPA plays autocrine and/or paracrine roles in the reproductive tract acting via various active LPARs.

### 5.2. LPA Influence on Estradiol (E_2_) Production and Follicle Stimulating Hormone (FSH) Action in Granulosa Cells of the Bovine Ovarian Follicle

In bovine follicles, Boruszewska et al. [81] were the first to demonstrate the influence of LPA on E_2_ synthesis and secretion in the granulosa cells (Figure 1). The authors documented that LPA and LPA together with FSH stimulated E_2_ production by cultured granulosa cells* in vitro* [81, Figure 1]. Since E_2_ promotes follicular development by regulating steroid production and the expression of gonadotrophin receptors in the bovine granulosa cells [88–90], Boruszewska et al. [81] presumed that LPA participated in ovarian follicle growth and differentiation. It has been well documented that E_2_ secretion is stimulated by FSH [91, 92], which acts by binding to specific, transmembrane FSH receptor (FSHR) [88, 89]. Boruszewska et al. [81] documented that LPA and LPA together with FSH stimulated FSHR gene expression in bovine granulosa cells.

Boruszewska et al. [81] also investigated the effect of LPA on the E_2_ synthesis pathway. Granulosa cells are able to convert thecal androgens to E_2_ by cytochrome P450 aromatase (CYP19A1) and 17*β*-hydroxysteroid dehydrogenase- (17*β*-HSD-) catalyzed reactions [88, 90, 92, 93]. In the study of Boruszewska et al. [81], LPA did not influence CYP19A1 transcript level, while treatment with LPA, FSH, and LPA together with FSH resulted in increased 17*β*-HSD mRNA expression in granulosa cells (Figure 1).

Concluding, LPA stimulates E_2_ production and FSH action in granulosa cells of the bovine ovarian follicle via increased expression of the FSHR and 17*β*-HSD genes, which in turn might account for the participation of LPA in ovarian follicle growth and differentiation.

### 5.3. The Action of LPA on Bovine CL during the Luteal Phase of Estrous Cycle and Early Pregnancy

**Figure 2:**
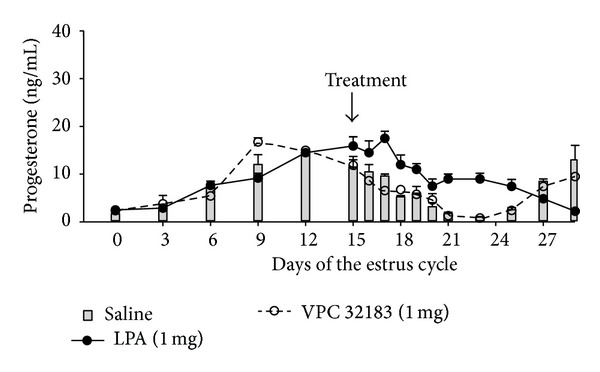
Concentrations of progesterone in peripheral blood plasma of heifers infused with saline (grey bars), LPA (1 mg; line), or LPA (1 mg) together with blocker of LPARs (VPC32183; 1 mg; dotted line) on day 15 of the estrous cycle. (Adapted from [40].)

In ruminants* in vivo* action of LPA was only examined in the cow [7, 40]. In these studies it was demonstrated that LPA administered into* aorta abdominalis* affected P4 and PG secretion during the luteal phase of the estrous cycle. The dose of 1 *μ*g LPA administered into the* aorta abdominalis* stimulated P4 and PGE_2_ concentration in the blood [7]. Woclawek-Potocka et al. [7] also showed that the inhibition of endogenous LPA action via the infusion of LPA1 receptor antagonist (Ki16425) caused the decrease of P4 and PGE_2_ concentrations, which suggested LPA influence on both endometrium and the CL. Moreover, Woclawek-Potocka et al. [40] found that in the heifers infused deeply into the* vagina*, near the* cervix* of the uterus with 1 mg LPA, spontaneous luteolysis was prevented, and the functional lifespan of the CL was prolonged in comparison with animals of the control group (Figure 2). The possibility of LPA action on P4 synthesis in the steroidogenic cells of the bovine CL was confirmed in the* in vitro* studies of Kowalczyk-Zieba et al. [16]. The authors found that LPA stimulated P4 secretion via stimulation of 3*β*-hydroxysteroid dehydrogenase/5Δ-4Δ isomerase (3*β*HSD) expression in steroidogenic CL cells [16].

We also found that LPA did not express only direct luteotropic action [16] but also indirect luteoprotective role inhibiting cytokine mediated regression of the bovine CL [83]. We examined the possibility of LPA-dependent modulation of tumor necrosis factor (TNF)*α* and IFN*γ* actions at the late luteal stage—when the luteolysing cytokines act the most. It has been documented before that TNF*α* together with IFN*γ* can serve as mediators of luteolytic actions of PGF_2*α*_ via inhibiting P4 production and stimulating apoptosis of the cultured bovine luteal cells [94–96]. Physiologically, in the organism, not only activated macrophages and lymphocytes produce TNF*α* and IFN*γ* but also fibroblasts and endothelial cells [97, 98]. Penny et al. [97] and Sakumoto et al. [99] demonstrated that total amount of TNF*α* and IFN*γ* rise significantly just after initiation of luteolysis, as the reason of a great amount of lymphocytes infiltrating the CL at this time. Moreover, Skarzynski et al. [100] demonstrated before that TNF*α* in low concentrations caused luteolysis (decreased P4 level), which could be augmented by various factors, including IFN*γ*. Concerning the possibility of LPA-dependent modulation of TNF*α* and IFN*γ* actions at the late luteal stage, Woclawek-Potocka et al. [83] demonstrated that LPA reversed the inhibitory effect of TNF*α* and IFN*γ* on P4 synthesis in the cultured bovine steroidogenic cells. These data are consistent with previous data obtained* in vivo* that LPA administered into* aorta abdominalis* or* intravaginally* increased P4 secretion in the cows during the luteal phase of the estrous cycle [7, 40]. In heifers, LPA-dependent prevention of the spontaneous luteolysis and prolongation of the functional lifespan of the CL* in vivo* were also reported before [40]. These results seem to be important because the midluteal stage represents a critical period in the CL lifespan for P4 secretion [101]. Woclawek-Potocka et al. [83] hypothesized that at the midluteal stage of estrous cycle, if the female becomes pregnant, that continued secretion of P4 from the CL can be directly supported by LPA or indirectly by reversing luteolyting action of TNF*α* and IFN*γ*.

Woclawek-Potocka et al. [83] also documented that LPA suppressed TNF*α*- and IFN*γ*-induced luteal cell apoptosis (Figure 1), which is known to occur during structural luteolysis [102, 103]. In the bovine CL it was demonstrated that LPA inhibited the stimulatory effect of TNF*α* and IFN*γ* on the expression of one of the mitochondrial regulatory proteins, Bax, which in turn orientates the cells towards the survival state [83]. In addition, apoptosis on the receptor level can also be initiated via TNF super family receptors (TNFRs). Sakumoto et al. [99] and Taniguchi et al. [96] demonstrated that TNF*α* induced apoptotic cell death of cultured bovine luteal cells mainly acting via TNFR1, whereas TNFR2 is the type of the receptor associated mainly with the prosurvival action of this cytokine in the organism [104]. In the study of Woclawek-Potocka et al. [83], LPA inhibited only the stimulatory effect of TNF*α* and IFN*γ* on TNFR1 expression in the cultured steroidogenic luteal cells on days 8–12 of the estrous cycle. The Fas antigen (Fas) also belongs to the TNF super family receptors which together with Fas ligand (FasL) transmit basic signals controlling intercellular apoptosis pathway [105]. Woclawek-Potocka et al. [83] found that in the presence of LPA, TNF*α* and IFN*γ* did not stimulate Fas and FasL expression in the cultured steroidogenic luteal cells on days 8–12 of the estrous cycle. Moreover, it has been documented before that the inhibition of intraluteal P4 action by various specific antagonists amplified Fas L-mediated apoptosis viathe increase of Fas and initiation of caspase (Casp)8 and Casp3 expressions as well as Casp3 activity in cultured bovine luteal cells [106]. High levels of Casp8 directly initiate cleavage of an effector Casp3, thereby initiating the execution phase of apoptosis [107]. During apoptosis executed through the mitochondrial pathway, active Casp8 stimulates the binding of proapoptotic Casp to mitochondria and inhibits association of antiapoptotic Bcl-2. This leads to the leakage of cytochrome c from the mitochondria into the cytosol, which in turn promotes formation of the apoptosome and triggers activation of the effector Casp3 [107]. In the bovine CL, LPA decreased cleaved Casp3 activity induced by TNF*α* and IFN*γ* [83]. However, in the bovine CL the onset of apoptosis is not observed until P4 production has declined [108, 109]. In this aspect Woclawek-Potocka et al. [83] surmised that in the bovine CL, in the presence of LPA, P4 secretion was supported and also TNF*α* and IFN*γ* could not induce apoptosis (Figure 1). Moreover, LPA reversed TNF*α*- and IFN*γ*-induced apoptosis via inhibition of the stimulatory effect of the cytokines on the expression of Bax, Fas-FasL system, TNFR1, and Casp3 activity in the cultured steroidogenic luteal cells, which orientated these cells towards the survival state [83].

**Figure 3:**
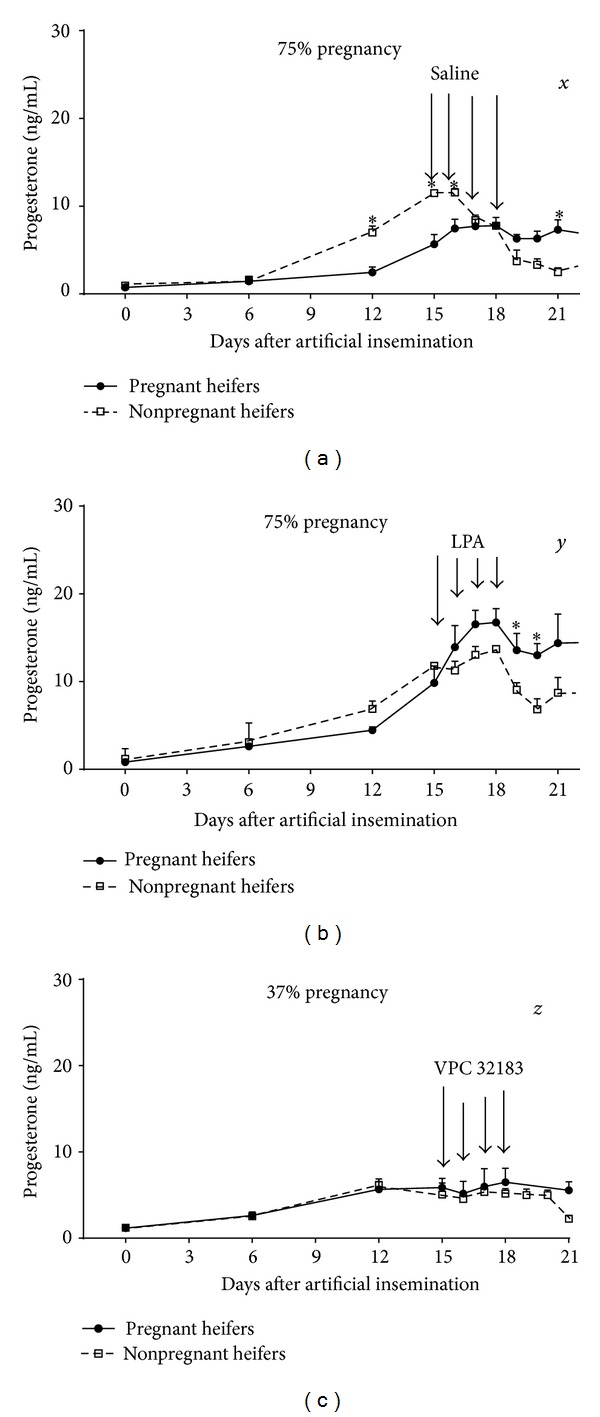
Concentrations of progesterone in peripheral blood plasma of pregnant (black dots) and nonpregnant (white squares) heifers infused with saline (a), 1 mg of LPA (b), or 1 mg of VPC32183 (c). All reagents were infused every 24 hours from day 15 to day 18 after insemination into the* vagina*. Different letters in the top right corner indicate significant differences (*P* < 0.05) between treated groups (*n* = 8 for each group of heifers). (Adapted from [40].)

The influence of LPA on early pregnancy in the cow was also examined [40]. Woclawek-Potocka et al. [40] demonstrated that LPA had strong effect on P4 and PGE_2_ secretion on days 15–18 of early pregnancy (Figure 3). Moreover, the authors proved that blocking the effect of endogenous LPA by administration of VPC32183 significantly decreased pregnancy rate compared with control and LPA-treated heifers [40, Figure 3]. LPA-induced PGE_2_ secretion* in vivo* may indirectly support CL function [110, 111] and have roles in establishing and maintaining pregnancy [112, 113]. Thus the authors suggested that LPA could be a luteoprotective factor in the bovine endometrium during both the estrous cycle and early pregnancy establishment in the cow [40]. The data obtained in the above studies seem to be important because the examined time frame (days 15–18) represent a critical period in the establishment of pregnancy. This is the time of the highest IFN*τ* production by the conceptus, just before implantation; therefore, the interactions between LPA and IFN*τ* cannot be excluded.

The interactions between LPA and IFN*τ* were studied* in vitro* by Kowalczyk-Zieba et al. [16] in bovine CL. The authors investigated whether LPA had a direct effect on P4 secretion from bovine luteal cells and whether it modulated IFN*τ* action in the luteal cells* in vitro* [16]. Kowalczyk-Zieba et al. [16] found that LPA stimulated P4 secretion from steroidogenic CL cells of the midluteal phase through stimulation of 3*β*HSD expression in these cells (Figure 1). These results are important because the midluteal stage represents a critical period in the CL lifespan for secretion of P4 [114]. Kowalczyk-Zieba et al. [16] hypothesized that at the examined time of estrous cycle, if the female becomes pregnant, continued secretion of P4 from the CL can be also supported by LPA. However, Kowalczyk-Zieba et al. [16] did not find any modulation of IFN*τ* action on P4 secretion in the luteal cells of the bovine CL. On the other hand, Kowalczyk-Zieba et al. [16] proved that LPA augmented IFN *τ*-dependent stimulation of ubiquitin-like IFN-stimulated gene 15 kDa protein (ISG15) and 2,5′-oligoadenylate synthase (OAS1) expression in the steroidogenic cells of the bovine CL (Figure 1). These two genes are expressed in the bovine CL of both cyclic and pregnant cows regardless of pregnancy status but are upregulated only during early pregnancy [115, 116].

The data obtained in cows prove that LPA can be an additional auxiliary luteotropic factor acting in the CL and in the endometrium during both the estrous cycle and early pregnancy establishment.

### 5.4. The Influence of LPA on PG Synthesis in the Bovine Endometrium

In ruminants, uterine PGs are crucial components in the regulation of estrous cycle and early pregnancy. Prostaglandin F_2*α*_ is the major luteolytic agent, whereas PGE_2_ has luteoprotective and antiluteolytic properties [111, 117]. Therefore, achieving an optimal PGF_2*α*_ to PGE_2_ ratio is essential for endometrial receptivity, maintenance of CL action, and P4 secretion as well as accurate pregnancy establishment [118]. The dynamic PG synthesis and action in the bovine endometrium [111, 117, 118] and possible interactions between LPA and PGs as well as mechanisms of LPA synthesis [119, 120] were well evidenced in the literature. Woclawek-Potocka et al. [7] tested the hypothesis, whether LPA signaling affected endometrial AA metabolism not only in rodents [120–122] and human [123] but also in cattle. In the bovine endometrium, positive correlation between LPAR1 and PGES expression at early pregnancy was demonstrated [7]. Moreover, LPAR1 expression was negatively correlated with the expression of PGFS during early pregnancy [7]. The authors claimed that these correlations explained that PGE_2_ and LPA act similarly and that PGF_2*α*_ and LPA act differently during early pregnancy in cow [7].

There are also data in the literature on the intracellular and enzymatic mechanisms of LPA- dependent stimulation of PG synthesis in the bovine endometrium [7, 40]. In the bovine uterus, LPA stimulated PGE_2_ synthesis via PGES mRNA stimulation only in stromal cells on days 8–10 and 16–18 of the estrous cycle and pregnancy [13, 40]. Moreover, LPA inhibited PGF_2*α*_ synthesis via PGFS mRNA stimulation only in epithelial cells on days 8–10 and 16–18 of pregnancy [13, 40]. Thus, Woclawek-Potocka et al. [13] suggested that LPA is an additional luteoprotective factor in the bovine endometrium during both the estrous cycle and early pregnancy. Since PGE_2_ stimulates CL function [110, 111] and has roles in establishing and maintaining pregnancy [112, 124], LPA, via stimulation of its synthesis, may be an important factor contributing to the establishment of pregnancy in the bovine endometrium. Woclawek-Potocka et al. [13] also suggested that this effect might be additionally augmented by LPA-dependent inhibition of PGF_2*α*_ synthesis during early pregnancy. The above data seem to be important, because the examined time frames are crucial phases during early pregnancy. First, days 8–10 represent the time of immunological pregnancy establishment as shown by Kelemen et al. [125], Barnea et al. [126], and Majewska et al. [127]. Moreover, days 8–10 after conception have recently been considered to be crucial in terms of early embryonic loss. In cattle, the major percentage of embryo loss occurs before day 16 following breeding, with some evidence pointing to greater losses before day 8 in high-producing dairy cows [128]. On the other hand second examined time frame—days 16–18 of early pregnancy represent the time of the highest IFN*τ* production by the conceptus, just before implantation. Therefore, the interactions between LPA and IFN*τ* at these phases cannot be excluded.

The data obtained in cows is consistent to a certain extent with data obtained in ovine trophectoderm cultured cells, in which LPA induced PGF_2*α*_ and PGE_2_ release [34]. However, the authors of this study excluded the possibility of the effect of LPA on PG release via changes in the mRNA expression of PGES and PGFS. Liszewska et al. [34] claimed that, in the case of trophectoderm cells, the phosphorylation of PLA2 by extracellular signal regulated kinase (ERK) is a critical step in the sequence of events leading to mobilization of AA, as was demonstrated previously by An et al. [129] in Jurkat T cells in humans. Liszewska et al. [34] hypothesized, that in trophectoderm cells, LPA-mediated phosphorylation of ERK may have caused rapid activation of PLA_2_ that resulted in a burst of PG synthesis independent of any modifications in gene expression. However, there are also reports in human and rats that LPA increased PGE_2_ synthesis in human monocytic and ovarian cancer cells [130, 131] as well as rat mesangial cells [132, 133] via upregulation of PTGS2. Moreover, in mice, targeted deletion of LPAR3 receptor resulted in implantation defects accompanied by a reduction in PTGS2 expression and the levels of PGE_2_ and PGI_2_ [120].

Despite different intracellular mechanisms of LPA-induced PG synthesis in the cow and ewe, a new biological function for LPA interaction with PGs in the contribution of pregnancy establishment in cows and in the regulation of the implantation process and embryonic development in ewes was designated in ruminants.

## 6. Conclusion and Future Perspectives

There is overwhelming evidence in many studies using a ruminant model that LPA signaling can have significant consequences for reproductive health. The effects of LPA depend on many various conditions such as the target tissue and physiological status of the animal as well as the phase of the estrous cycle or pregnancy. However, the most important issue connected with LPA signaling is the fact that there is the possibility of LPA synthesis directly in the area of the reproductive tissues. Therefore, it is crucial to examine carefully the effects of this biologically active compound on reproductive outcomes using animal models that can the most closely mimic reproductive processes in human.

In spite of many limitations in conducting well-designed human studies, information gathered from already published ones combined with the large number of the studies already available in ruminants, clearly demonstrate that LPA has the ability to influence the reproductive performance of an adult female.
